# Intermittent fasting two days versus one day per week, matched for total energy intake and expenditure, increases weight loss in overweight/obese men and women

**DOI:** 10.1186/s12937-022-00790-0

**Published:** 2022-06-04

**Authors:** Paul J. Arciero, Karen M. Arciero, Michelle Poe, Alex E. Mohr, Stephen J. Ives, Autumn Arciero, Molly Boyce, Jin Zhang, Melissa Haas, Emma Valdez, Delaney Corbet, Kaitlyn Judd, Annika Smith, Olivia Furlong, Marley Wahler, Eric Gumpricht

**Affiliations:** 1 Human Nutrition and Metabolism Laboratory, Department of Health and Human Physiological Sciences, Skidmore College, 815 North Broadway, Saratoga Springs, NY 12866 USA; 2 College of Health Solutions, Arizona State University, Phoenix, AZ 85004 USA; 3 Isagenix International LLC, Gilbert, AZ 85297 USA

**Keywords:** Intermittent fasting, Protein pacing, Weight loss, Fat mass, Fat-free mass, Hunger, Insulin-like growth factor -1 (IGF-1)

## Abstract

**Background:**

Intermittent fasting (IF), consisting of either a one-day (IF1) or two consecutive days (IF2) per week, is commonly used for optimal body weight loss. Our laboratory has previously shown an IF1 diet combined with 6d/week of protein pacing (P; 4–5 meals/day evenly spaced, ~ 30% protein/day) significantly enhances weight loss, body composition, and cardiometabolic health in obese men and women. Whether an IF1-P or IF2-P, matched for weekly energy intake (EI) and expenditure (EE), is superior for weight loss, body composition, and cardiometabolic health is unknown.

**Methods:**

This randomized control study directly compared an IF1-P (*n* = 10) versus an IF2-P (*n* = 10) diet on weight loss and body composition, cardiovascular (blood pressure and lipids), hormone, and hunger responses in 20 overweight men and women during a 4-week weight loss period. Participants received weekly dietary counseling and monitoring of compliance from a registered dietitian. All outcome variables were assessed pre (week 0) and post (week 5).

**Results:**

Both groups significantly reduced body weight, waist circumference, percent body fat, fat mass, hunger, blood pressure, lipids, glucose, and increased percent fat-free mass (*p* < 0.05). However, IF2-P resulted in significantly greater reductions in body weight (-29%) and waist circumference (-38%) compared to IF1-P (*p* < 0.05), and showed a strong tendency for greater reductions in fat mass, glucose, and hunger levels (*p* < 0.10) despite similar weekly total EI (IF1-P, 9058 ± 692 vs. IF2-P, 8389 ± 438 kcals/week; *p* = 0.90), EE (~ 300 kcals/day; *p* = 0.79), and hormone responses (*p* > 0.10).

**Conclusions:**

These findings support short-term IF1-P and IF2-P to optimize weight loss and improve body composition, cardiometabolic health, and hunger management, with IF2-P providing enhanced benefits in overweight women and men.

**Trial registration:**

This trial was registered March 03, 2020 at www.clinicaltrials.gov as NCT04327141.

**Supplementary Information:**

The online version contains supplementary material available at 10.1186/s12937-022-00790-0.

## Background

Intermittent fasting (IF) is an increasingly popular and effective dietary strategy for weight loss (WL) and improved health. Numerous recent meta-analyses and reviews confirm this contention [1–5], although the benefits are not universal [6], and perhaps due to insufficient lengths of sustained fasting (> 16 h)***.*** Of the myriad types of IF, several are particularly common and have shown modest efficacy on WL and health improvement: a) “periodic” IF includes fasting one (IF1; < 36 h) to two (IF2; > 48 h) days/week and feeding ad libitum (eating freely) the remaining five or six days [7]; b) alternate-day fasting (ADF), consisting of no (or very low) energy (calorie) intake every second day while eating ad libitum the other days [8]; and c) time-restricted eating (TRE) consisting of fasting for 12–20 h/day and the remaining time-consuming calories freely [9]. The IF-mediated benefits are associated with increased oxidation of fatty acids (lipolysis) and ketone body formation (ketogenesis), activated cell-signaling pathways (insulin sensitivity, reduced inflammation, autophagy), and preservation of lean body mass, known as “metabolic switching” [1–11]. Interestingly, these mechanisms are typically not fully activated until at least 24 h of fasting [5]. Although a common feature of IF diets emphasizes the timing and quantity of calories eaten (or not eaten) during the feasting/fasting days, there is also an under-emphasis on the “quality” of calories consumed during the feeding and/or fasting period. This lack of emphasis on the quality of nutrient-dense (vitamins, minerals, antioxidants, botanicals, unprocessed, high-quality protein, etc.) energy consumed during feasting and fasting periods is a significant oversight. Indeed, this concept warrants further study, especially given the proven benefits of high-quality nutrient-dense food intake on improving weight management and overall health.

Our lab has conducted a series of experiments focused on low-sugar, nutrient-dense calorie intake during short- and long-term weight loss interventions, with and without IF protocols, in overweight/obese adults [12–14]. A consistent finding of these studies is the profound impact that low-sugar, nutrient-dense calorie intake, including meal replacement bars and shakes, has on WL and body composition management and health outcomes. Specifically, we have shown “protein pacing” (P, 4–6 meals/day evenly-spaced, 25–40 g protein/meal, > 30% protein/day) combined with a one-day (36 h) nutrient-dense IF (IF1-P) meal pattern significantly improves body composition and cardiometabolic health [12–15]. A low-sugar (< 60 g/day) IF1-P diet incorporates timed-daily ingestion of protein-rich meals (P), from meal replacements, supplementation, and whole food sources, providing approximately 0.25–0.4 g/kg body weight protein per meal (or > 30% protein per day; 1500–1800 kcals/day) combined with nutrient-dense (antioxidant/botanical) beverages with high protein/fiber low-sugar snacks on IF days (350–500 kcals/day) [13–15].

Despite the demonstrated benefits of both traditional IF and nutrient-dense IF-P regimens, there is little research comparing the effects of extended (> 24 h) IF1-P versus an IF2-P meal pattern, matched for total weekly kcals, macronutrient distribution, and physical activity energy expenditure, on body weight and composition, hormones, and hunger ratings in overweight and obese men and women during short-term (4 weeks) WL. Thus, the primary purpose of this study was to examine body composition, and cardiometabolic responses to short-term (weeks 0–4) nutrient-dense IF1-P (36 h IF) or IF2-P (60 h IF) dietary interventions. Based on previous research, we hypothesized that a short-term (4 weeks) IF2-P diet intervention would result in greater WL, and improved body composition and cardiometabolic health compared to an IF1-P regimen in overweight/obese men and women.

## Methods

### Participants

This study enrolled 200 individuals from the Saratoga Springs, NY area. Potential participants responded to flyers, local newspapers, or emails advertising the study. The number of subjects initially screened was 55, of which 42 were eligible for participation. Participants were healthy, nonsmoking, overweight/obese men and women. A comprehensive medical examination/history assessment was performed by their physicians to rule out any current cardiovascular or metabolic disease. For at least six months before the start of the study, all subjects were either sedentary or lightly active (< 30 min, two days/week of organized physical activity), overweight or obese (BMI > 27.5 kg/m^2^; % body fat > 30%), weight stable (± 2 kg), and middle-aged (30–65 years). Every participant provided informed written consent in accordance with the Skidmore College Human Subjects review board before participation. The study was approved by the Human Subjects Institutional Review Board of Skidmore College (IRB#: 1911–859). All experimental procedures were performed in adherence with related New York State regulations and the Federal Wide Assurance, consistent with the National Commission for the Protection of Human Subjects of Biomedical and Behavioral Research, and in agreement with the Helsinki Declaration (revised in 1983). This trial was registered at clinicaltrials.gov as NCT04327141.

### Experimental design

#### Study timeline

**Fig. 1:**
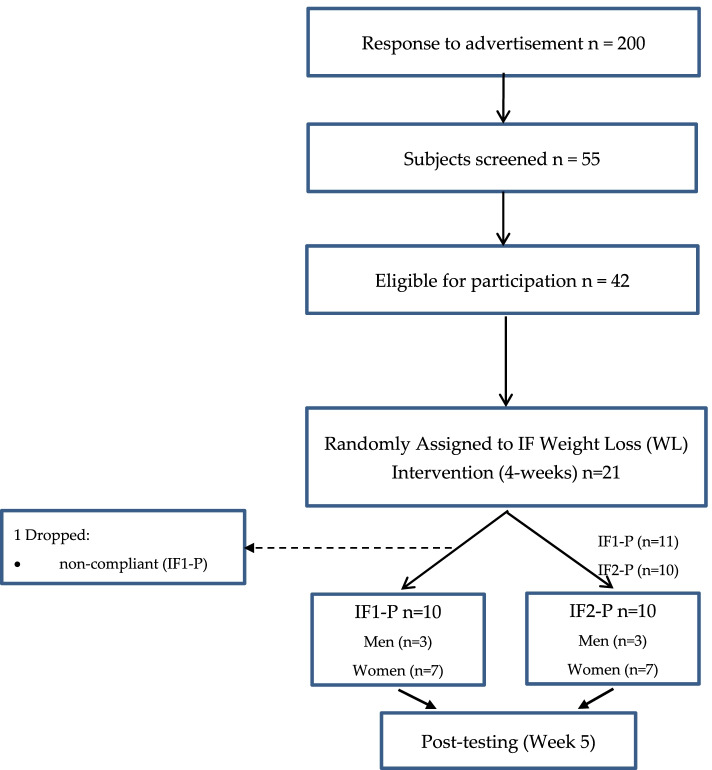
CONSORT flow diagram for the study

Subjects were enrolled in two separate cohorts due to COVID-19 restrictions regarding personnel laboratory access, such that half enrolled in fall 2020 and the other half in spring 2021. The current study includes a subgroup of a more extensive intervention study comparing intermittent fasting and protein pacing diet (IF-P, *n* = 20) versus a heart-healthy (HH, *n* = 19) diet on body composition, cardiometabolic, microbiome, and metabolomic outcomes over eight weeks in 39 overweight/obese women and men. Therefore, only necessary comparisons between the IF-P groups will be presented in this manuscript. The 20 IF-P study participants were matched for weight and BMI and randomly assigned to either: a) an intermittent fasting diet for one day/week (IF 36 h total) and protein pacing (P) diet for the remaining six days/week (IF1-P), or b) an IF diet for two consecutive days (IF 60 h total) and P for the remaining five days/week (IF2-P) for four weeks. (Fig. 1 shows the CONSORT study flow chart). Complete blinding was not possible given the study design, as both groups consumed identical weekly total calorie intakes and macronutrient distributions (including food and nutritional supplements as described in detail below (see lines 174–214 and Supplemental Table S1) over the entire 4 weeks. In addition, both groups received identical nutritional support from the registered dietitian during weekly meetings. Lastly, participants in both groups were not explicitly made aware of study participants in the other group. The primary justification for using a 4-week intervention was to allow for comparisons to previously published interventions. More importantly, this period is sufficient for physiological and biochemical induced pathways to be expressed and subsequent quantification of changes in all outcome measures following a calorie restricted/IF dietary protocol. Extending beyond 4-weeks reduces compliance and may be overly excessive for a caloric restriction and 2 day IF and create undue metabolic, physiologic, hormonal, and psychological stress in the study participants.

**Fig. 2:**
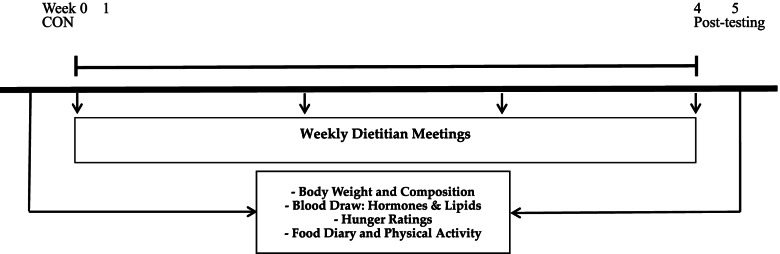
Study Timeline for testing during IF-P study.CON, conrol, week 0; Post-testing, week 5

All laboratory testing procedures (see below) were performed at baseline control (CON, week 0) and week 5 (Fig. 2).

The weight loss (WL) phase began with all participants following a four-week controlled IF-P intervention, as detailed below. During the 1-week baseline control (CON), subjects maintained a stable body weight by consuming a similar caloric intake as their pre-enrollment caloric intake while maintaining their sedentary lifestyle. Following CON baseline testing, participants were provided with detailed instructions on their WL dietary guidelines and scheduled weekly 1-h meetings with a registered dietitian.

#### Dietary interventions

### Weight loss (WL) (weeks 1–4): intermittent fasting (IF) – protein pacing (P) diets

Participants were randomly assigned to one of two different IF-P protocols beginning at week one and instructed to follow the meal plan for four weeks.

### IF day diets

IF1-P consisted of ~ 400 kcals per day in which participants were provided a variety of supplements and snacks. Specifically, participants consumed each of the following supplements mixed with either cold or hot water: an adaptogen/antioxidant-rich beverage four servings, evenly spaced throughout the day (morning, noon, mid-afternoon, evening; Cleanse for Life®, 160 kcals/day); two servings of an antioxidant beverage (morning/evening; Ionix® Supreme, 40 kcals/day); one serving of a collagen bone broth beverage (dinner meal; Collagen Bone Broth, 45 kcals/day); one serving of an electrolyte beverage (AMPED™ Hydrate, 20 kcals/day); and for the dinnertime meal, one serving of snack crackers (dinner meal; Harvest/Whey Thins™, 100 kcals/day) with the bone broth. Once these basic requirements were met, participants were able to choose from a list of “options” to achieve a total of ~ 400 kcals/day, these included: half of a dark chocolate square (IsaDelight® Chocolates, 30 kcals/day); one to two servings of an anti-oxidant/caffeinated beverage (BEA™, 20 kcals/day); ½ of a nut bar (Snack Bites, 50 kcals/day); or a combination of fresh vegetables/fruits and nuts/seeds (not to exceed 50 kcals/day) as recommended by the research team to support their energy needs. IF2-P followed an identical meal pattern for both IF days, except for consuming an additional 100 kcals from the “options” list to achieve ~ 500 kcals/day for each of the two consecutive fasting days. Sample menus and meal timing for IF1-P and IF2-P intermittent fasting (IF) days are shown in Supplemental Table S1. All participants were provided a detailed cookbook including recipes and menus for the dinner meal and afternoon snacks that met the requirements for IF and P macronutrient and total kcal intakes.

### P day diets

IF1-P consumed a protein pacing (P) diet consisting of four and five meals/day for women and men, respectively; two of which (breakfast and evening) were liquid meal replacement shakes with added whole foods (Whole Blend IsaLean® Shakes, 350/400 kcals, 30/36 g of protein/meal); a whole food evening dinner meal (450/500 kcals men), an afternoon snack (200 kcals, men only), and an evening protein snack (Whole Blend IsaLean® Shakes or Bars; 200 kcals). This dietary regimen provided 1350 and 1700 kcals/day for women and men, respectively, and a macronutrient distribution targeting 35% protein, 35% carbohydrate, and 30% fat. This macronutrient distribution has previously been used successfully in our lab to induce an energy deficit without compromising lean body mass [12, 13]. IF2-P followed a similar P meal protocol with the following modifications: breakfast/evening shakes increased to 400/450 kcals, women and men, respectively; and evening snack added 50 kcals to the evening snack from whole food “options” (250 kcals total). This dietary regimen provided 1500 and 1850 kcals/day for women and men, respectively, and similar macronutrient distribution and total weekly calorie intakes (~ 8500 kcals/week) as IF1-P. Isagenix International, LLC (Gilbert, AZ, USA) provided all meal replacement shakes, bars, beverages, and supplements. Sample menus and meal timing for IF1-P and IF2-P protein pacing (P) days are shown in Supplemental Table S1.

### Compliance

All subjects met with a registered dietitian weekly during WL to facilitate healthy eating habits and adherence to the respective dietary protocols. In addition, subjects were provided detailed written instructions for each IF diet plan. They were closely monitored through daily participant-researcher communication (e.g., email, text, and mobile phone), two-day food diary analysis, weekly dietary intake journal inspections, distribution of weekly meal/supplement containers, and return of empty packets and containers. Weekly meetings were held via ZOOM (Zoom Video Communications, Qumu Corporation, San Jose, CA, USA) for each IF group separately, with the dietitian and the research team to verify compliance with the dietary meal plans, clarify dietary guidelines, and answer questions. The overall compliance rate in each group was high (> 90%), which was defined as consuming more than 90% of their respective meals/supplemented feedings. Noncompliance was defined as being absent from more than two consecutive dietitian meetings and under- or over-consuming ≥ three inappropriate meal/supplement servings a week for ≥ two consecutive weeks at a time. Two-day food records were completed by every participant at two different time points (Week 0 and 4) to further verify compliance to each IF diet (see Energy Balance Assessment, below).

#### Laboratory testing procedures

### Body composition assessments

At weeks 0 and 5, all participants were tested between the hours of 6:00 a.m. and 9:00 a.m., after an overnight fast, and underwent body composition assessments (height, body weight, and total body composition). Body weight was obtained using a standard digital scale and, height was obtained without shoes using a stadiometer. Waist circumferences, in centimeters (cm) were obtained with a standard tape measure placed around the waist two centimeters above the iliac crest by the same investigator (K.M.A.), at each time point. Body composition was assessed by BODPod (Cosmed, Chicago, IL, USA) for the measurement of total fat mass (FM), % body fat (%BF), fat-free mass (FFM), % FFM (body weight/FFM). Standard body mass index (BMI) measurements were obtained by dividing the subject’s weight (kg) by the square of their height (m^2^).

### Energy balance assessment

Energy balance was calculated for each individual by closely monitoring both physical activity energy expenditure (EE) (Actigraph LLC, Pensacola, FL, USA) as well as their energy intake (EI) for two days during baseline control (CON, week 0) and week 4 (1 day each of IF and P). The registered dietitian and a member of the research team instructed participants on completing detailed dietary food records of portion sizes and food items. All food logs were recorded using the Food Processor SQL Edition (version 11.6.522 ESHA Research, Salem, OR, USA, 2012). A single trained research team member (M.P.) and the PI (P.J.A.) analyzed all the food logs to reduce inter-investigator variation. Each participant was also given a checklist to help them adhere to the IF regimens. Participants were asked to maintain their current level of physical activity (sedentary/low activity) and to abstain from starting any new exercise programs throughout the entire WL intervention. To verify sedentary/low activity levels, all participants wore an Actigraph Data Analysis Software accelerometer (v. 6.13.3; Actigraph LLC, Pensacola, FL, USA) around their waist for two days during weeks 0 and 4. All EE accelerometer data were analyzed by the same research team member (M.P.) for all participants.

### Cardiovasculare and plasma biomarkers

Blood pressure and heart rate was obtained with an automated blood pressure monitor (Omron Healthcare Inc., Milton Keynes, UK) following > 15 min of quiet sitting. For plasma hormone measurements, 12-h fasted venous blood samples (~ 20 mL) were collected into EDTA-coated vacutainer tubes and centrifuged (Hettich Rotina 46R5) for 15 min at 2500 rpm at -4 °C. After separation, plasma was stored at − 80 °C until analyzed. Plasma concentrations of insulin (INS), ghrelin (GRL), and glucagon-like polypeptide -1 (GLP-1) were analyzed using ELISA’s (RayBiotech, Peachtree Corners, GA, USA). Glucagon and insulin-like growth factor -1 (IGF-1) were analyzed by custom-plex immunoassays (Eve Technologies Corporation, Calgary, AB Canada) and, blood glucose and lipids were determined using a colorimetric assay (Cholestech LDX Analyzer, Abbott Laboratories, Abbott Park IL, USA). Test–retest intraclass correlation (*r*) and coefficient of variation (CV) in our laboratory with *n* = 15 was: insulin, and glucose (mg/dL) *r* = 0.95, CV = 3.2%, and *r* = 0.97, CV = 5.3%, respectively.

### Feelings of hunger and satiety

Visual analog scales (VAS) were administered at weeks 0 and 5 to evaluate the effects of the IF-P protocols on hunger, satiation, quantity-of-food-to-eat, and desire-to-eat. Briefly, participants were instructed, using a pen and paper, to mark their levels of hunger, satiety, quantity-of-food-to-eat, and desire-to-eat on a 100 mm line that was anchored at either end with “0” (none) to “100” (extreme). For each of these measures, the degree of sensation was quantified by the distance from the “0” mm point. All VAS scoring was measured by the same investigator (M.P.).

### Statistical analysis

Statistical analysis was performed using SPSS software (Ver. 27; IBM-SPSS, Armonk NY, USA). Before starting the study, the sample size was determined through power analysis based on the primary outcome variables body weight and body composition to achieve an effect size of 0.25 with 80% power at alpha 0.05 based on previous data [12, 16, 17]. This analysis determined that *n* = 10 was required to detect a significant mean difference of 1.4 kg weight loss between the two diet intervention groups (IF1-P vs. IF2-P) during WL. Absolute changes in body weight (kg) and composition, biomarkers, and hunger ratings were calculated. Two way (2 × 2) factorial mixed model Analysis of Variance (ANOVA) were performed for WL parameters using IF-P (IF1-P vs. IF2-P) and time (week 0 vs. 5) to determine the main effects. Data analysis was not performed blinded but each intervention group was assigned a number code. A per-protocol approach was used on data for all compliant study participants and an intent-to-treat analyses was performed on data (pre and post) from all randomized study participants. The per-protocol analysis is presented in the results and the ITT analysis is presented in the Supplemental Table S2. One-tailed tests were utilized for this study, and the significance was set at *p* < 0.05. All values are reported as means ± standard error (SE) unless stated otherwise.

## Results

### Weight loss (WL; weeks 0–4)

#### Subject characteristics

One individual in IF1 did not adhere to the dietary guidelines and was dropped due to non-compliance. ITT analysis was conducted including this individual and is presented in Supplemental Table S2). Thus, descriptive baseline characteristics of the twenty subjects (14 women and six men) who completed WL are reported in Table 1. Both groups were similar for all variables at baseline.

**Table 1:** Baseline (week 0) characteristics of participants for WL

Variable	IF1-P (*n* = 10)	IF2-P (*n* = 10)
Age (years)	47.3 ± 10.0	52.0 ± 8.6
Height (cm)	166.4 ± 12.7	172.8 ± 10.0
Weight (kg)	86.9 ± 18.5	99.4 ± 25.6
Body fat (%)	38.2 ± 7.4	42.0 ± 8.2
Body mass index (kg/m^2^)	31.3 ± 5.1	33.6 ± 9.7
Waist circumference (cm)	98.0 ± 9.8	108.9 ± 17.8
Systolic blood pressure (mmHg)	123 ± 18	124 ± 14
Diastolic blood pressure (mmHg)	84 ± 9	86 ± 6
Resting heart rate (bpm)	75 ± 12	69 ± 7

Values are means, Standard error *SE*

### Dietary intake and physical activity during WL

The IF-P WL diet interventions significantly altered both groups’ dietary energy and macronutrient intake (Table 2).

**Table 2:** Changes in dietary intake and physical activity during WL

**Variable**		**IF1-P (*n* = 10)**	**IF2-P (*n* = 10)**
Energy (kcals/day)	Pre	2452 ± 166	2483 ± 149
	Post ^a^	1458 ± 113	1459 ± 87
Energy (kcals/week)	Pre	17,166 ± 1165	17,384 ± 1046
	Post ^a^	9058 ± 692	8389 ± 438
Protein (%)	Pre	15 ± 1	17 ± 1
	Post ^a^	34 ± 2	35 ± 3
Protein (g)	Pre	94 ± 8	105 ± 11
	Post ^a^	119 ± 10	126 ± 12
Fat (%)	Pre	39 ± 3	39 ± 3
	Post ^a^	34 ± 1	28 ± 2
Fat (g)	Pre	104 ± 8	110 ± 10
	Post ^a^	54 ± 6	47 ± 4
Carbohydrate (%)	Pre	42 ± 2	43 ± 3
	Post ^a^	32 ± 1	37 ± 5
Carbohydrates (g)	Pre	257 ± 24	268 ± 26
	Post ^a^	119 ± 17	116 ± 11
Sodium (mg)	Pre	3456 ± 344	3197 ± 440
	Post ^a^	1402 ± 71	1655 ± 243
Fiber (g)	Pre	20 ± 3	19 ± 3
	Post ^a^	32 ± 3	27 ± 2
Sugars (g)	Pre	100 ± 17	107 ± 15
	Post ^a^	43 ± 10	29 ± 4
Physical Activity (kcals/day)	Pre	285 ± 87	295 ± 57
	Post	207 ± 31	299 ± 47

Values are means, Standard error *SE*

^a^ Significant time effect (Pre vs. Post), *p* < 0.05

Specifically, total energy intake following IF-P diets decreased significantly (*p* < 0.05) in both groups by ~ 40% (1000 kcals/day) during WL, with no differences between groups. This reduction was due to significant (*p* < 0.05) decreases in percentage and grams of dietary fat (5%-10%; 50–60 g) and carbohydrate (7%–10%; 138–152 g) intake, as well as increased percentage and amount of protein intake (17%; 21–25 g, respectively; *p* < 0.05) in both groups. In addition, IF-P protocols significantly (*p* < 0.05) increased dietary fiber (8–12 g/day) and decreased sugar (57–77 g/day) and sodium (1500–2000 mg/day) intake. Both groups maintained similar amounts of physical activity energy expenditure throughout the WL period.

### Body weight and composition during WL

Effects of the IF-P protocol on body weight and body composition are shown in Table 3.

**Table 3:** Changes in body weight and composition during WL

**Variable**		**IF1-P (*n* = 10)**	**IF2-P (*n* = 10)**
Body weight (BW, kg)	Pre	86.9 ± 5.9	99.4 ± 8.1
	Post ^a,b^	82.3 ± 5.4	92.3 ± 7.2
Waist Circumference (WC, cm)	Pre	98.0 ± 3.1	108.9 ± 5.6
	Post ^a,b^	93.1 ± 3.4	100.9 ± 6.1
Body Mass Index (BMI, kg/m^2^)	Pre	31.3 ± 1.6	33.6 ± 3.1
	Post ^a,c^	29.6 ± 1.4	31.2 ± 2.8
Total Body Fat (%BF, %)	Pre	38.2 ± 2.3	42.0 ± 2.6
	Post ^a^	36.2 ± 2.6	40.0 ± 2.4
Fat Mass (FM, kg)	Pre	33.0 ± 3.3	41.6 ± 2.6
	Post ^a,c^	29.7 ± 3.2	37.0 ± 4.3
Fat-Free Mass (FFM, kg)	Pre	53.1 ± 4.0	56.5 ± 4.4
	Post ^a^	51.9 ± 3.9	54.6 ± 4.1
FFM/BW (%)	Pre	61.8 ± 2.3	58.0 ± 2.6
	Post ^a^	63.8 ± 2.6	60.0 ± 2.4

Values are means, *SE*

^a^ Significant time effect (Pre vs. Post), *p* < 0.05

^b^ Significant time x group effect (IF1-P vs. IF2-P; Pre vs. Post), *p* < 0.05

^c^ Trend for time x group effect, *p* < 0.10

**Fig. 3:**
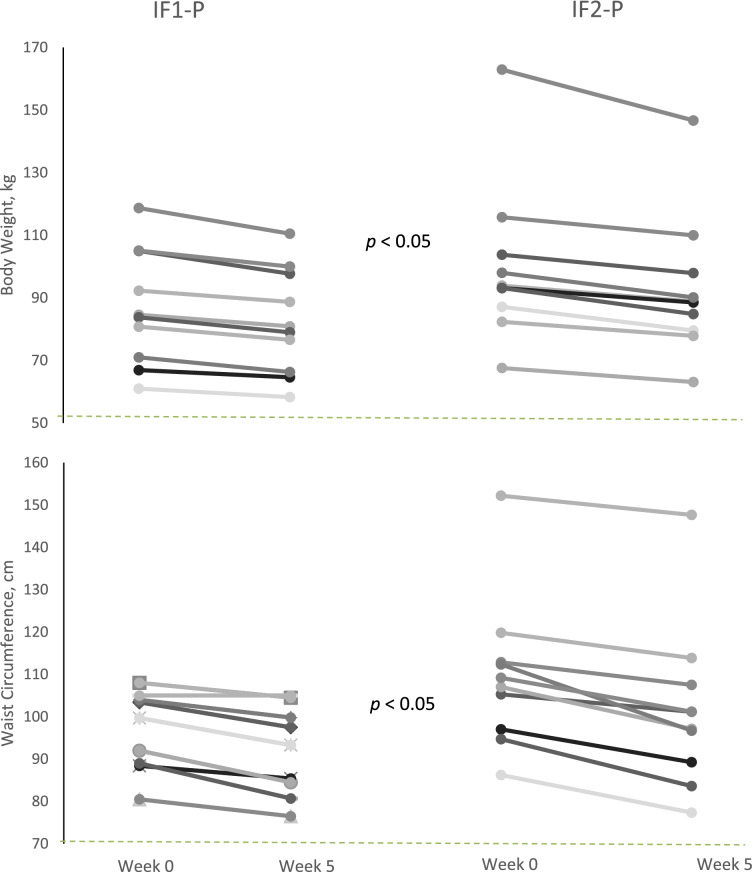
Individual changes in body weight and waist circumference during WL between IF1-P and IF2-P

Relative to baseline, both IF-P groups had significant reductions (*p* < 0.05) in all outcome measures (body weight, waist circumference, BMI, total body fat %, fat mass, and fat-free mass), however, IF2-P resulted in greater (*p* < 0.05) body weight and waist circumference loss compared to IF1-P (Fig. 3). IF1-P and IF2-P lost 4.7 kg and 7.1 kg or 5.2% and 7% of body weight from baseline, respectively (*p* < 0.05). Similarly, IF1-P and IF2-P reduced waist circumference by 5 cm and 7.6 cm or 4.8% and 8% from baseline, respectively (*p* < 0.05). Of special note, when expressed as a percentage of body weight, FFM increased by 2%, with no differences between groups.

### Plasma hormones during WL

Plasma biomarkers are reported in Table 4 and show no significant change from baseline or between groups. This observation suggests that hormone changes were not responsible for the body composition and cardiometabolic responses between IF1-P and IF2-P during the 4-week intervention.

**Table 4:** Changes in plasma hormones during WL

**Variable**		**IF1-P (*n* = 10)**	**IF2-P (*n* = 10)**
Glucose (mg/dL)	Pre	88.8 ± 4.3	98.3 ± 5.9
	Post ^a^	90.0 ± 3.6	92.2 ± 2.8
Insulin (μU/mL)	Pre	10.9 ± 0.8	9.9 ± 0.8
	Post ^b^	10.4 ± 0.7	9.5 ± 0.8
Ghrelin (pg/mL)	Pre	352.6 ± 66.1	430.6 ± 53.9
	Post	391.5 ± 37.9	404.2 ± 35.1
Glucagon (pg/mL)	Pre	37.0 ± 5.6	46.3 ± 7.1
	Post	41.4 ± 4.1	48.5 ± 5.3
GLP-1 (pg/mL)	Pre	1815.8 ± 638.3	1404.3 ± 236.1
	Post	2487.2 ± 1034.7	1175.6 ± 258.9
IGF-1 (ng/mL)	Pre	52.6 ± 4.7	41.9 ± 5.3
	Post ^c^	59.4 ± 4.2	40.8 ± 4.8

Values are means, *SE*

^a^ Significant time effect (Pre vs. Post), *p* < 0.05

^b^ Trend for time x group effect, *p* < 0.10

^c^ Trend for time effect *p* < 0.10

### Cardiovascular responses during WL

Both groups experienced significant reductions in blood pressure (SBP, 8/5 mmHg; DBP, 6/3 mmHg; IF1-P / IF2-P, respectively, *p* < 0.05), fasting total cholesterol, LDL-C, and triglycerides (17%/16%; 10%/14%, and 21%/11%; IF1-P and IF2-P, respectively, *p* < 0.01), with no differences between groups during the 4-week WL intervention (Table 5). HDL-C decreased significantly in both groups; however, the TC:HDL-C remained unchanged by the dietary interventions.

**Table 5:** Changes in blood pressure and lipids during WL

**Variable**		**IF1-P (*n* = 10)**	**IF2-P (*n* = 10)**
Systolic Blood Pressure, SBP (mmHg)	Pre	123 ± 6	124 ± 4
	Post ^a^	114 ± 5	119 ± 5
Diastolic Blood Pressure, DBP (mmHg)	Pre	84 ± 3	86 ± 2
	Post ^a^	77 ± 3	84 ± 3
Total Cholesterol (mg/dL)	Pre	184.9 ± 12.6	180.1 ± 8.8
	Post ^a^	153.7 ± 11.5	153.7 ± 12.4
LDL-C (mg/dL)	Pre	106.8 ± 12.3	109.4 ± 7.9
	Post ^a^	96.3 ± 12.1	95.1 ± 9.9
HDL-C (mg/dL)	Pre	54.6 ± 5.1	51.2 ± 4.6
	Post ^a^	41.9 ± 3.5	42.6 ± 4.0
Triglycerides (mg/dL)	Pre	117.7 ± 25.7	101.5 ± 24.7
	Post ^a^	80.4 ± 9.6	77.5 ± 10.8
TC:HDL (mg/dL)	Pre	3.7 ± 0.5	3.9 ± 0.5
	Post	4.0 ± 0.5	3.8 ± 0.4

Values are means, *SE*

^a^ Significant time effect (Pre vs. Post), *p* < 0.05

### Hunger ratings during WL

Self-reported feelings of hunger, desire to eat, the quantity of food to eat, and fullness are reported in Table 6. Both groups experienced significant reductions in desire to eat (44%) and quantity of food to eat (30%), with a tendency of hunger ratings to be lower in IF2-P (40%) compared to IF1-P (*p* = 0.10).

**Table 6:** Changes in hunger ratings during WL

**Variable**		**IF1-P (*n* = 10)**	**IF2-P (*n* = 10)**
Hunger (mm)	Pre	29.4 ± 6.6	43.2 ± 6.2
	Post ^b,c^	28.1 ± 5.6	25.7 ± 5.7
Desire to Eat (mm)	Pre	43.0 ± 7.4	42.7 ± 7.2
	Post ^a^	23.9 ± 4.8	25.5 ± 5.9
Quantity to Eat (mm)	Pre	47.1 ± 6.7	46.5 ± 6.8
	Post ^a^	33.0 ± 4.9	33.5 ± 5.9
Fullness (mm)	Pre	44.4 ± 5.6	56.7 ± 7.4
	Post	49.9 ± 5.4	47.6 ± 6.7

Values are means ± SE

^a^ Significant time effect (Pre vs. Post), *p* < 0.05

^b^ Trend for time x group effect, *p* < 0.10

^c^ Trend for time effect *p* < 0.10

## Discussion

The primary aim of this study was to compare short-term IF – one day ((IF1-P, 36 h) per week vs. two days (IF2-P, 60 h) per week on body weight and composition, and cardiometabolic responses in overweight/obese women and men. We observed that both IF1-P and IF2-P produced significant weight loss accompanying reductions in waist circumference and body fat. This weight loss was also associated with reductions in blood lipids, blood pressure, and desire and quantity-of-food-to-eat measures. Comparatively, IF2-P yielded greater weight loss and reduction in waist circumference than IF1-P, despite similar reductions in total energy intake (-1000 kcals/day) and unchanged levels of energy expenditure (200–300 kcals/day) and similar increases in fat-free mass.

These favorable effects appear independent of alterations in circulating hormones, which remained largely unchanged throughout the WL period.

### Body weight and composition

Our finding of significant weight loss and improved body composition outcomes with IF agrees with recent meta-analyses and reviews [2, 5–11]**,** although this is not a universal finding [6–18]. Some data also suggest a substantial loss of lean body mass may occur with IF depending on the length and degree of fasting [6, 18] which is a common observation during weight loss [18]. However, in our study, the proportion of FFM increased more than 2%, with a total FFM loss of only ~ 1 kg, or less than 20% of the total weight lost. This favorable response is likely due to the emphasis on high-quality protein pacing feedings (whey and plant-based). During an energy deficit, muscle protein synthesis is reduced [19]**,** and higher protein intake may attenuate this reduction or even increase MPS [20–22]. Our laboratory has consistently demonstrated favorable fat mass and attenuated loss or retention of lean body mass in overweight individuals engaged in energy restriction or IF interventions [12, 13].

Explanations for the greater weight and waist circumference loss and body fat reductions with IF2-P may be attributed to several molecular and cellular adaptations associated with intermittent fasting. First, as characterized in detail by Anton et al. [5], intermittent fasting induces a “metabolic switch” with fuel utilization and nutrient partitioning selectively transitioning from glucose to fat oxidation. With prolonged fasting of 60 h in IF2 subjects’ metabolism likely further progressing from lipolysis to ketogenesis [23]. Mechanistically, the additional day of fasting per week in IF2 participants may have likely elevated ketone production, which may further activate AMP-activated protein kinase (AMPK), a key energy-sensing modulator within the cell [24].

The current findings are the first to compare two short-term IF regimens combined with protein pacing on anthropometric and cardiometabolic measures. The novelty of these findings is underscored by three distinct factors: 1) matching weekly total energy intake, including macronutrient distribution and energy expenditure in both groups; 2) the clearly defined length of IF between groups (36 versus 60 h); and 3) consumption of nutrient-dense energy intake during the IF and protein pacing (P) days. Most previous research examined IF according to the length of time or the quantity of food allowed during the fasting period, with less emphasis on the quality of the energy (calories) consumed during fasting and feeding days. The current study provided the unique opportunity to directly quantify the effects of two different fasting periods on body composition in a group of overweight women and men.

### Plasma biomarkers

Neither IF protocol significantly affected plasma hormones, glucose, or insulin levels; however, the IF2-P group trended towards lower plasma glucose, and both groups trended towards lower plasma insulin. Our findings agree with several recent IF interventions that report plasma glucose levels are tightly regulated and relatively unresponsive to IF [9–25]. In contrast, IF interventions typically result in reductions in plasma insulin [7]. Indeed, our previous intervention [13], which included an IF-1 dietary pattern during WL, noted reductions in both plasma glucose (approximately 10%) and insulin (approximately 40%). Potential contributing factors accounting for differences between the two studies include the significantly greater level of WL of subjects in the previous study compared to this one (11.6 kg vs. 5.8 kg) and the duration of the WL phases (12 weeks vs. four weeks). Finally, it must be noted that our intervention included a low carbohydrate/sugar intake which generally does not impact plasma insulin concentrations [26], and that reduction in insulin levels may not occur during short-term interventions [27].

### Cardiovascular responses

Both IF interventions significantly reduced lipid concentrations and blood pressure. These findings agreed with our previous study [13] and others [28–30]. HDL-C also decreased during WL, supporting other observations [27]; however, this reduction did not affect the TC: HDL-C ratio. The hypotriglyceridemic effect of IF has also been noted in some [28–31] but not all studies [32]. Blood pressure reductions in response to IF have also been reported by many researchers [27–29, 33, 34], although these reductions are typically associated with longer interventions.

### Hunger ratings

Both IF1 and IF2 significantly reduced self-reported desire and quantity-of-food-to-eat. Interestingly, feelings of hunger and satiety were unchanged. This finding was surprising considering that participants experienced a 40% reduction in total energy intake (primarily from carbohydrate and fat; protein intake modestly increased) compared to baseline. In contrast, acute and longer-term energy restriction in overweight and obese individuals has been reported to increase appetite and appetite-associated hormones [35–38]. Therefore, the current study’s results have important compliance implications for future recommendations as an effective weight loss strategy.

Macronutrient composition and energy intake shifted dramatically relative to dietary protein and fiber intake. In line with the protein leverage hypothesis, increasing protein density may promote satiety, which can aid in preventing energy overconsumption and obesity [39]. Dietary fiber and the resistant starches present in many of the supplements utilized in this study also have positive satiety and satiety-related hormone effects [40, 41].

As noted above, there was a trend for a greater reduction in hunger during IF2. This observation was intriguing considering the longer consecutive fasting duration and a more significant decrease in body weight and waist circumference. In addition, both groups had similar weekly energy intake and expenditure and the objective measures of appetite, including ghrelin (hunger-associated hormone) and GLP-1 (fullness-associated hormone), remained unchanged from baseline levels. Other fasting research incorporating calorie restriction has reported similar findings in obese adults using IF [42, 43] and ADF regimens [44, 45]. As noted previously, we postulate IF2-P participants experienced enhanced ketogenesis compared to IF1-P participants, and previous research suggests a complex ketone-mediated suppression of some measures of hunger or appetite [46]. Finally, our findings may be mediated through gut microbiome alterations as the gut-brain axis is emerging as an important regulator of appetite and food reward signaling [47], an understudied and nascent topic of intense investigation [48].

### Strengths and limitations

Strengths of the present study include: (a) direct comparison within and between interventions; (b) careful weekly monitoring and counseling with a registered dietitian; (c) direct measurement of physical activity by accelerometry; (d) high compliance (> 95%); and, perhaps most interesting, (e) the extent of the weight loss during the COVID-19 pandemic in early fall of 2020 through the spring of 2021. Indeed, recent literature has highlighted the adverse impact of COVID-19 on weight [49, 50]. We are also aware of several limitations For example, one of the IF2-P participants may have strongly influenced the group’s response. Moreover, a non-compliant participant from IF1-P was dropped in the presented results. Per our ITT analysis, including this individual obscured the significant interaction effect for BW. Importantly, this individual significantly restricted their dietary intake (< 2 SD, mean calorie intake) which contrasts with the normal non-compliance issues associated with caloric restriction clinical research. We contend the presented per-protocol results are more reflective and accurate of the intended intervention as this individual invariably produced an additional weight loss effect due to their severe caloric restriction which impacted the group mean and statistical analysis. The greater weight loss with IF-2 vs IF-1 may have been mediated by increased resting metabolism, which was not measured in the current study. Our previous investigation [13] did not observe an increased resting metabolic rate in response to 12-weeks of IF1-P in obese men and women, and therefore, this consideration requires further investigation. Also, circulating ketones were also not measured. Unfortunately, this was not feasible in the current study due to scheduling conflicts and stringent COVID laboratory restrictions preventing time-sensitive analysis following a fasting day. Finally, the short-term design of our study does not allow speculation about long-term benefits, compliance, or adverse responses to the dietary protocol.. As the study was a subgroup analysis, additional research is necessary to confirm these findings in free-living overweight/obese adults following an IF-P over more extended intervention periods.

## Conclusions

Our results support the combination of intermittent fasting and protein pacing as effective short-term nutritional interventions for weight loss and improvements in body composition. These changes facilitate additional cardiometabolic benefits without adversely impacting hunger or appetite. Although our study design cannot distinguish between benefits afforded to intermittent fasting versus protein pacing, both likely contribute to significant, perhaps independent benefits. A long-term investigation of this nutritional eating pattern for addressing obesity and cardiovascular health is warranted.

## Supplementary Information

**Additional file 1: Table S1.**Sample menus and meal timing for Protein Pacing (P) and Intermittent Fasting (IF) Days for (IF1-P) and (IF2-P) Study Participants during 4 Week Weight Loss (WL). **Table S2.** Intention-to-Treat (ITT) analysis including the non-compliant participant on primary outcomes.

## Data Availability

The datasets used and/or analysed during the current study are available from the corresponding author on reasonable request.

## Abbreviations

IF: Intermittent fasting
P: Protein Pacing
HH: Heart healthy
WL: Weight loss
FFM: Fat-free mass
FM: Fat mass
%BF: Percent body fat
BMI: Body mass index
%FFM: Percent fat-free mass
TC: Total cholesterol
TG: Triglycerides
SBP: Systolic blood pressure
DBP: Diastolic blood pressure
IGF: 1-Insulin-like growth factor -1
