# Supplementary material for: Intermittent fasting two days versus one day per week, matched for total energy intake and expenditure, increases weight loss in overweight/obese men and women
Source: Nutr J. 2022 Jun 4;21:36. doi: 10.1186/s12937-022-00790-0 (PMC9166203; doi:10.1186/s12937-022-00790-0)
Supplement: Supplementary file 1 — Additional file 1: Table S1.Sample menus and meal timing for Protein Pacing (P) and Intermittent Fasting (IF) Days for (IF1-P) and (IF2-P) Study Participants during 4 Week Weight Loss (WL). Table S2. Intention-to-Treat (ITT) analysis including the non-compliant participant on primary outcomes. [file 12937_2022_790_MOESM1_ESM.docx]

**Table S1. Sample menus and meal timing for Protein Pacing (P) and Intermittent Fasting (IF) Days for (IF1-P) and (IF2-P) Study Participants during 4 Week Weight Loss (WL)**

| **Variable** | **IF1-P** | | **IF2-P** | |
| --- | --- | --- | --- | --- |
|  | **Men**  **(1700 kcals/day)** | **Women**  **(1350 kcals/day)** | **Men**  **(1850 kcals/day)** | **Women**  **(1500 kcals/day)** |
| Breakfast  (0600–0800) | Liquid meal replacement shake; 400 kcals, **36 g protein**, 40 g carbohydrate, 12 g fat; Caffeine beverage;  Antioxidant & Adaptogen mix;  20 kcals | Liquid meal replacement shake; 350 kcals, **30 g protein**, 35 g carbohydrate, 10 g fat; Caffeine beverage;  Antioxidant & Adaptogen mix;  20 kcals | Liquid meal replacement shake; 450 kcals, **36 g protein**, 43 g carbohydrate, 15 g fat; Caffeine beverage;  Antioxidant & Adaptogen mix;  20 kcals | Liquid meal replacement shake; 400 kcals, **30 g protein**, 43 g carbohydrate, 12 g fat; Caffeine beverage;  Antioxidant & Adaptogen mix;  20 kcals |
| Lunch (1100–1300) | Choice of liquid meal replacement shake or Fresh vegetables with choice of fish/poultry/ beef/plant-based protein; 400 kcals, **36 g protein**, 40 g carbohydrate, 12 g fat | Choice of liquid meal replacement shake or Fresh vegetables with choice of fish/poultry/ beef/plant-based protein; 350 kcals, **30 g protein**, 35 g carbohydrate, 10 g fat | Choice of liquid meal replacement shake or Fresh vegetables with choice of fish/poultry/ beef/plant-based protein; 400 kcals, **36 g protein**, 40 g carbohydrate, 12 g fat | Choice of liquid meal replacement shake or Fresh vegetables with choice of fish/poultry/ beef/plant-based protein; 350 kcals, **30 g protein**, 35 g carbohydrate, 10 g fat |
| Mid-Afternoon snack (1400–1600) | Greek yogurt, fruit; 200 kcals, **20 g protein**; 15 g carbohydrate; 5 g fat |  | Greek yogurt, fruit; 200 kcals, **20 g protein**; 15 g carbohydrate; 5 g fat |  |
| Dinner (1700–1900) | Fish/Poultry/Beef, fresh vegetables, chopped nuts, dried fruit, olive oil, milk; 500 kcals, **36 g protein**; 50 g carbohydrate; 18 g fat | Fish/Poultry/Beef, fresh vegetables, chopped nuts, dried fruit, olive oil, milk; 450 kcals, **30 g protein**;  50 g carbohydrate; 15 g fat | Fish/Poultry/Beef, fresh vegetables, chopped nuts, dried fruit, olive oil, milk; 500 kcals, **36 g protein**; 50 g carbohydrate; 18 g fat | Fish/Poultry/Beef, fresh vegetables, chopped nuts, dried fruit, olive oil, milk; 450 kcals, **30 g protein**;  50 g carbohydrate; 15 g fat |
| Evening snack (2100–2200) | Protein shake/bar; 225 kcals, **30 g protein**; 20 g carbohydrate;  7 g fat | Protein snack; 200 kcals, **25 g protein**; 20 g carbohydrate;  7 g fat | Protein snack; 275 kcals, **30 g protein**; 20 g carbohydrate;  9 g fat | Protein snack; 250 kcals, **25 g protein**; 20 g carbohydrate;  9 g fat |

**Example of Nutrient-Dense Intermittent Fasting Day Intake for IF1-P and IF2-P During WL**

| Antioxidant plant-based powder | 4/day | 160 kcal total |
| --- | --- | --- |
| Plant-based herbal adaptogen powder | 2/day | 40 kcal total |
| Collagen bone broth | 2/day | 45 kcal total |
| Low-glycemic protein crackers | 1/day | 100 kcal total |
| Electrolyte beverage | 1/day | 20 kcal total |
| Optional foods: | 1/day | Dark chocolate square; antioxidant/caffeine beverage; ½ nut bar; fresh vegetable/fruit, nut/seed mix (all < 50 kcal total for IF1-P and 150-200 kcal total for IF2-P) |

**Table S2. Intention-to-Treat (ITT) analysis including the non-compliant participant on primary outcomes.**

| Variable |  | IF1-P (*n* = 11) | IF2-P (*n* = 10) |
| --- | --- | --- | --- |
| Body weight (BW, kg) | Pre | 89.4 ± 5.8 | 99.4 ± 8.1 |
|  | Post ^a^ | 84.3 ± 5.3 | 92.3 ± 7.2 |
| Waist Circumference (WC, cm) | Pre | 100.1 ± 3.5 | 108.9 ± 5.6 |
|  | Post ^a,b^ | 95.1 ± 3.6 | 100.9 ± 6.1 |
| Body Mass Index (BMI, kg/m^2^) | Pre | 31.9 ± 1.6 | 33.6 ± 3.1 |
|  | Post ^a^ | 30.1 ± 1.4 | 31.2 ± 2.8 |
| Total Body Fat (%BF, %) | Pre | 38.4 ± 2.1 | 42.0 ± 2.6 |
|  | Post ^a^ | 36.4 ± 2.3 | 40.0 ± 2.4 |
| Fat Mass (FM, kg) | Pre | 34.2 ± 3.2 | 41.6 ± 2.6 |
|  | Post ^a^ | 30.5 ± 3.0 | 37.0 ± 4.3 |
| Fat-Free Mass (FFM, kg) | Pre | 54.4 ± 3.8 | 56.5 ± 4.4 |
|  | Post ^a^ | 52.9 ± 3.6 | 54.6 ± 4.1 |
| FFM/BW (%) | Pre | 60.9 ± 2.1 | 58.0 ± 2.6 |
|  | Post ^a^ | 63.1 ± 2.3 | 60.0 ± 2.4 |

Values are means ± SE; ^a^ Significant time effect (Pre vs. Post), *p* < 0.05. ^b^ Significant time x group effect (IF1-P vs. IF2-P; Pre vs. Post), *p* < 0.05.
